# Infections Following Skin Graft Surgery in Australia: Incidence, Graft Type and Anatomical Site Associations and Microbiology

**DOI:** 10.3390/idr18050111

**Published:** 2026-10-02

**Authors:** Semun Galimam, Naleesha Habib, Madhumitha Sangeetham Krishnan Asokumar, Ruchir Chavada

**Affiliations:** 1 Pharmacy Department, Calvary Mater Hospital, Newcastle, NSW 2298, Australia; 2 Surgical Department, Prince of Wales Hospital, Sydney, NSW 2031, Australia; 3 Geriatrics Department, Gosford Hospital, Gosford, NSW 2250, Australia; 4 Microbiology & Infectious Diseases, NSW Health Pathology, Central Coast, Gosford, NSW 2250, Australia

**Keywords:** surgical site infection, skin graft, antimicrobial stewardship, microbiology, postoperative infection, antibiotic prophylaxis

## Abstract

**Background/Objectives**: Infection following skin grafting can compromise graft take and prolong hospitalisation, but contemporary data describing infection incidence, microbiology and graft-specific associations are limited. We aimed to determine the incidence and microbiology of microbiologically confirmed postoperative skin graft infection, examine associations with graft type and anatomical site and describe antimicrobial prescribing practices. **Methods**: We conducted a retrospective cohort study of 977 skin graft procedures performed in 770 patients at two regional hospitals in New South Wales, Australia, between July 2021 and August 2024. Infection was defined as a clinician-diagnosed postoperative skin graft infection with microbiological confirmation of a pathogenic organism. Associations between graft type/site and infection were explored using patient-clustered modified Poisson regression. Patient comorbidities and wound-contamination status were not consistently available and were not analysed as covariates. **Results**: A total of 66 microbiologically confirmed infections occurred among 977 procedures (6.8%; 95% CI 5.3–8.5%). Median length of stay was longer in infected than non-infected cases (34 vs. 3 days; *p* < 0.001). Split-thickness grafts involving the foot, toe and extensive granulating areas had higher observed infection incidence, while full-thickness nasal grafts had lower observed incidence; subgroup estimates were imprecise. *Staphylococcus aureus* (47.0%) and *Pseudomonas aeruginosa* (22.7%) were the predominant organisms. In the nested antimicrobial audit of 119 encounters involving 116 patients, peri-operative prophylaxis and postoperative antibiotic prescribing were common. **Conclusions**: This Australian cohort provides contemporary estimates of microbiologically confirmed infection following skin grafting and identifies graft type/site patterns that may inform future prospective investigation. Interpretation is limited by the retrospective design, microbiological confirmation requirement, sparse subgroup events and unavailable patient- and wound-level confounders; therefore, the observed associations should not be interpreted as independent risk factors or evidence for specific preventive or treatment strategies.

## 1. Introduction

Skin grafts (SGs), including split-thickness skin grafts (SSGs) and full-thickness skin grafts (FTSGs), are widely used in reconstructive and plastic surgery for trauma, burns, chronic wounds and oncological defects [1]. Infection following grafting can compromise graft take, contribute to graft loss, prolong hospitalisation and increase healthcare costs [2,3]. Although surgical site infections (SSIs) are well described across many surgical specialities [4], contemporary large-cohort data specifically characterising infection following skin graft surgery remain limited, particularly in Australian inpatient settings.

The risk of infection and graft failure is likely influenced by a combination of patient-, wound- and procedure-related characteristics. The previous literature has identified factors including patient comorbidity, anatomical site and local wound characteristics as potentially relevant to postoperative wound infection [5]. Graft type and thickness may also influence graft survival because SSGs and FTSGs differ in their biological characteristics and requirements for revascularisation [1,6,7]. In addition, the condition and microbiology of the recipient wound bed may be important; for example, the presence of *Pseudomonas aeruginosa* has been associated with poorer SSG outcomes in chronic venous leg ulcers [8]. However, the available literature is heterogeneous in population, indication, anatomical site and outcome definition, limiting direct comparison between studies.

The microbiology of postoperative skin graft infection is also incompletely characterised. Although *Staphylococcus aureus* and *Pseudomonas aeruginosa* are important causes of wound and healthcare-associated infection [9,10], their relative contribution to microbiologically confirmed infection following skin grafting has not been clearly defined in large contemporary cohorts. Similarly, evidence regarding antimicrobial prophylaxis in skin graft surgery remains limited, with uncertainty regarding which patients or procedures, if any, derive benefit from prophylactic antimicrobial therapy [11,12]. Contemporary data integrating infection incidence, graft type and anatomical site, microbiology and antimicrobial prescribing may therefore assist in defining priorities for future preventive and antimicrobial-stewardship research.

The primary objectives of this study were to quantify the incidence of microbiologically confirmed postoperative skin graft infection, characterise the associated microbiology and explore whether infection incidence differed according to graft type and anatomical site. We hypothesised that infection incidence would vary across graft type–site categories. A secondary objective was to describe antimicrobial prescribing practices in a nested hospital encounter-level audit cohort.

## 2. Materials and Methods

### 2.1. Study Design and Population

We conducted a retrospective observational cohort study of 977 skin graft procedures performed at Wyong and Gosford Hospitals (Central Coast Local Health District, NSW, Australia) between 1 July 2021 and 13 August 2024. All adult and paediatric patients undergoing grafting during the study period were eligible. Procedures were identified from institutional operative records, discharge summary diagnosis codes and electronic medical records. Microbiological information was obtained from electronic medical records and laboratory systems.

Cases were defined as graft procedures complicated by post-operative graft site infection meeting the predefined outcome definition. Where patients underwent multiple graft procedures, each procedure was analysed as a separate observation because infection risk was considered procedure-specific.

**Figure 1:**
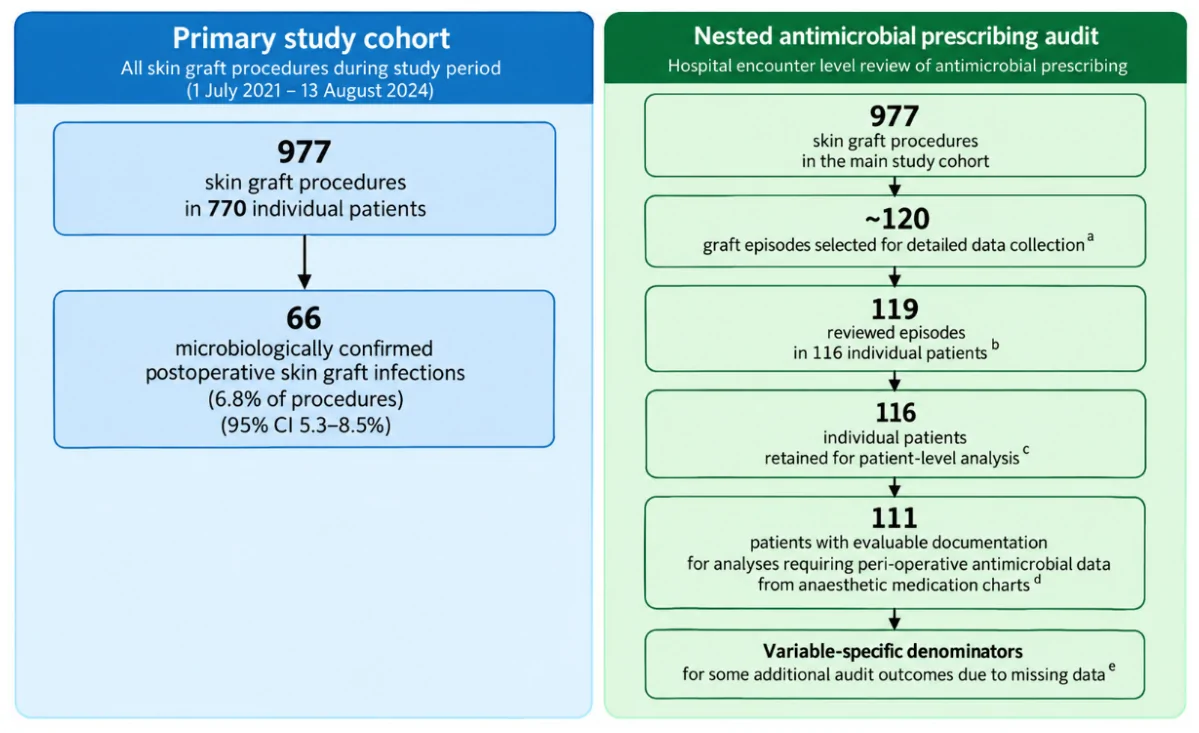
Study cohort and nested antimicrobial prescribing audit flow diagram. ^a^ The nested audit included both split-thickness and full-thickness skin graft procedures. Given the resource-intensive nature of manual medical-record review, approximately 120 graft episodes were selected for detailed data collection. ^b^ The resulting dataset comprised 119 episodes involving 116 individual patients; three patients had more than one eligible episode. ^c^ Where patients had multiple eligible surgical procedures, only the earliest procedure was retained for patient-level analysis, resulting in 116 individual patients. ^d^ Five patients were excluded from analyses requiring anaesthetic medication chart data because the relevant handwritten charts could not be reliably interpreted, leaving 111 patients with evaluable documentation. ^e^ Denominators for individual variables were smaller where data were missing.

To further characterise postoperative antimicrobial management, a nested hospital encounter-level antimicrobial prescribing audit was undertaken (Figure 1). The nested audit included both split-thickness and full-thickness skin graft procedures. Given the resource-intensive nature of manual medical-record review, approximately 120 graft episodes were selected for detailed data collection. Selection was not contingent on the presence of infection, antimicrobial exposure, microbiological sampling or graft failure, allowing both uncomplicated and potentially infected graft episodes to be evaluated. Detailed review included peri-operative and postoperative antimicrobial therapy, microbiological sampling and results, dressing type, infectious diseases consultation, graft outcome and clinical response. The resulting nested audit dataset comprised 119 episodes involving 116 individual patients. For analyses requiring peri-operative antimicrobial data from anaesthetic medication charts, five patients were excluded because the relevant handwritten charts could not be reliably interpreted, leaving 111 patients with evaluable documentation. The audit was descriptive in nature and was not intended to evaluate prescribing appropriateness, guideline adherence or the effectiveness of antimicrobial therapy.

### 2.2. Ethics and Governance

This project was classified as a clinical audit and conducted using routinely collected data under local institutional governance processes. Formal Human Research Ethics Committee approval was not required. Individual patient consent was not required for this retrospective audit. Medical record numbers were removed from the collected data prior to analysis.

### 2.3. Outcomes

The primary outcome was postoperative skin graft infection defined as a clinician-diagnosed graft-site infection with microbiological confirmation—a positive culture from a graft-site specimen such as a wound swab, aspirate or tissue. No predefined postoperative surveillance interval was specified. Infections were included when they were clinically documented as being related to the graft procedure during the available postoperative follow-up and met the microbiological confirmation criteria. Where multiple cultures were obtained for a single infection episode, organisms were de-duplicated at the episode level.

Secondary outcomes were length of hospital stay, microbiological profile of infections and associations between graft type and anatomical site with infection. In the antimicrobial audit cohort, additional outcomes included peri-operative prophylaxis, postoperative antibiotic prescribing, microbiological sampling, directed therapy, infectious diseases consultation, length of antimicrobial therapy and infection follow-up.

### 2.4. Microbiological Analysis 

Clinical microbiological specimens were processed by the routine pathology laboratories at the participating centres. Polymicrobial infections were included in the microbiological analysis. Where multiple organisms were isolated from a single infection episode, each organism was counted separately. The percentage of infected procedures in which each organism was identified was calculated using the 66 microbiologically confirmed infected procedures as the denominator. Because polymicrobial infections contributed to more than one organism category, these percentages were not mutually exclusive and did not sum to 100%. Bacterial isolates were identified using matrix-assisted laser desorption/ionisation time-of-flight mass spectrometry (MALDI-TOF MS; Bruker Diagnostics, Billerica, MA, USA) with flexControl software versions 3.4.204.10 and 3.4.207.20 and flexAnalysis version 3.4.79.0. Antimicrobial susceptibility testing was performed using the VITEK^®^ 2 automated microbiology system (bioMérieux, Marcy-l’Étoile, France) (software versions 9.02, 9.03.3, 9.03.4 and 9.04.4) with susceptibility interpretation undertaken in accordance with the laboratory’s standard operating procedures and contemporary clinical EUCAST breakpoints.

### 2.5. Data Collection and Statistical Analysis

Data were extracted from electronic medical records, operative records and laboratory information systems. Variables included graft type, anatomical site, microbiology results and length of stay (LOS). Age/date of birth and sex were not included in the predefined dataset and therefore demographic characteristics, including age, sex and paediatric/adult distribution, could not be reported. Details on comorbidities such as diabetes, wound class, contamination status and peri-operative antibiotic prophylaxis were not consistently available in the main cohort and were therefore not analysed as covariates.

Microbiologically confirmed infection incidence was estimated using exact (Clopper–Pearson) 95% confidence intervals. Associations between graft characteristics and infection were assessed using chi-square testing and modified Poisson regression with robust standard errors clustered at the patient level to estimate relative risks and 95% confidence intervals. Small SSG Other Site, the largest graft category, was used as the reference group to provide a stable comparator. This category comprised small split-thickness skin grafts to anatomical sites not represented by the predefined site-specific categories and was therefore heterogeneous. Categories with no infection events were excluded from regression analyses because stable relative risk estimates could not be obtained. Given the limited number of infection events and sparse data across graft subgroups, analyses were restricted to univariable models and should be considered exploratory.

Analyses were conducted at the procedure level because postoperative infection was considered a procedure-specific outcome. Patient-level clustering was used to account for within-patient correlation arising from patients contributing multiple graft procedures.

LOS was defined as the duration of the hospital admission during which the graft procedure was performed (index admission). For patients undergoing multiple graft procedures within a single admission, the same LOS was assigned to each procedure. LOS was analysed at the procedure level using negative binomial regression to account for overdispersion. As procedures from the same patient were not independent, this approach does not account for within-patient clustering and may overestimate precision; thus, results should be interpreted as exploratory. In addition, infection may have occurred during the admission, so the observed association between infection and LOS may reflect both prolonged hospitalisation resulting from infection and the greater opportunity for infection to occur during longer admissions.

Statistical significance was defined as a two-sided *p* < 0.05. Organism-specific regression analyses for *S. aureus* and *P. aeruginosa* were also explored, but several graft categories were excluded due to perfect prediction and estimates were unstable because of sparse data.

## 3. Results

### 3.1. Infection Incidence

A total of 977 skin graft procedures were performed in 770 individual patients during the study period. Sixty-six procedures met the study definition for microbiologically confirmed postoperative skin graft infection, corresponding to an incidence of 6.8% (95% CI 5.3–8.5%) (Table 1). Procedures were analysed as the unit of observation because postoperative infection was considered a procedure-specific outcome. As some patients contributed multiple graft procedures during the study period, within-patient correlation was accounted for in the graft type and anatomical site analyses using robust standard errors clustered at the patient level.

### 3.2. Length of Stay

Hospitalisation was substantially longer among infected cases. The median LOS was 34 days (IQR 13–52) among infected procedures and 3 days (IQR 0–13) among non-infected procedures. LOS differed significantly according to infection status in the negative binomial regression (*p* < 0.001) (Table 2). As noted above, this association may reflect both prolonged hospitalisation attributable to infection and the greater opportunity for infection to occur during longer admissions.

### 3.3. Graft Type and Anatomical Site Associations with Infection

Using “Small SSG Other Site” as the reference category, full-thickness grafts to the nose were associated with a lower observed incidence of infection (RR 0.093, 95% CI 0.013–0.688; *p* = 0.020). Full-thickness grafts to the face were not significantly associated with infection after accounting for patient-level clustering (RR 0.205, 95% CI 0.028–1.502; *p* = 0.119). Higher observed infection incidence was identified for small split skin grafts to the foot (RR 3.107, 95% CI 1.430–6.749; *p* = 0.004), split skin grafts of extensive granulating areas (RR 4.439, 95% CI 1.421–13.862; *p* = 0.010) and small split skin grafts to the toe (RR 5.548, 95% CI 1.315–23.407; *p* = 0.020) (Table 3). These estimates should be interpreted cautiously because several subgroup event counts were small and confidence intervals were wide.

Relative risks were estimated using modified Poisson regression with robust standard errors clustered at the patient level. Estimates should be interpreted as exploratory because of sparse infection events in several graft categories.

### 3.4. Microbiology

Microbiological isolates are summarised in Table 4. Among microbiologically confirmed infections, *Staphylococcus aureus* accounted for 47.0% and *Pseudomonas aeruginosa* for 22.7%.

Across all procedures, *S. aureus* was isolated in 31 of 977 procedures (3.2%, 95% CI 2.2–4.5%) and *P. aeruginosa* in 15 of 977 procedures (1.5%, 95% CI 0.9–2.5%). Other organisms were less frequent, including *Escherichia coli* in 7 procedures (0.7%, 95% CI 0.3–1.5%) and Group B streptococci in 5 procedures (0.5%, 95% CI 0.2–1.2%). *Acinetobacter baumannii*, *Enterobacter cloacae*, *Enterococcus faecium*, *Granulicatella adiacens*, *Haemophilus influenzae*, *Morganella morganii*, *Proteus mirabilis*, group A streptococci, group D streptococci and *Yokenella regensburgei* were each isolated in five or fewer cases and collectively accounted for a minority of infections.

Methicillin-resistant *S. aureus* accounted for 8/31 (25.8%) of *S. aureus* isolates and Vancomycin-resistant *Enterococcus* was identified in a single case. Of the 66 microbiologically confirmed infections, 59 (89.4%) were monomicrobial and 7 (10.6%) were polymicrobial. Of the seven polymicrobial infections, five involved two organisms and two involved three organisms.

### 3.5. Organism–Graft Type Associations

Organism-specific regression analyses were limited by sparse events across graft categories, with only 31 procedures involving *S. aureus* and 15 involving *P. aeruginosa*. Several graft categories contained no organism-specific events, resulting in unstable or non-estimable regression estimates. Accordingly, no robust graft type–specific associations with *S. aureus* or *P. aeruginosa* infection could be identified. The available data were insufficient to determine whether graft type was associated with *S. aureus* or *P. aeruginosa* infection.

### 3.6. Antimicrobial Prescribing Practices in a Nested Audit Cohort

The resulting nested audit dataset initially comprised 119 episodes involving 116 individual patients. Where patients had multiple eligible surgical procedures, only the earliest procedure for each patient was retained for analysis to ensure that each patient contributed a single observation, resulting in a patient-level dataset of 116 patients. For analyses requiring peri-operative antimicrobial data from anaesthetic medication charts, five patients were excluded because the relevant handwritten charts could not be reliably interpreted, leaving 111 patients with evaluable documentation. Peri-operative antibiotic prophylaxis was administered in 89.2% of cases (99/111), with cefazolin as a single preoperative dose being the most commonly used regimen. Postoperative antibiotics were prescribed in 68.5% of cases (76/111), with cephalexin being the most frequently prescribed agent for 5–7 days.

Microbiological sampling was performed in 36.0% of cases (40/111), while directed antimicrobial therapy following culture results occurred in 10.8% of cases (12/111). Infectious diseases consultation was documented in 7.3% of cases (8/109), reflecting minor missing data for this variable. The median length of antimicrobial therapy was 5 days (IQR 0–5).

Among 106 cases with assessable infection follow-up, 7 infections occurred (6.6%) (Table 5). Postoperative antibiotic prescribing was not associated with infection (4/73 [5.5%] vs. 3/33 [9.1%]; RR 0.60, 95% CI 0.14–2.54; *p* = 0.675), although the analysis was likely underpowered and no conclusions regarding treatment effectiveness can be drawn from these observational data.

Infection was more frequently identified among patients who underwent microbiological sampling than among those who did not (6/39 [15.4%] vs. 1/67 [1.5%]; RR 10.31, 95% CI 1.29–82.50; *p* = 0.0096). Similarly, infection was more frequent among patients who received an infectious diseases consultation than among those who did not (3/8 [37.5%] vs. 4/97 [4.1%]; RR 9.09, 95% CI 2.45–33.77; *p* = 0.0090). These associations likely reflect clinical responses to suspected or established infection rather than effects of microbiological sampling or infectious diseases consultation on infection risk.

## 4. Discussion

In this large two-site regional hospital cohort, microbiologically confirmed postoperative skin graft infection occurred in 6.8% of procedures and was associated with substantially longer LOS. Reported infection rates following skin grafting range from approximately 3% to 10% depending on patient population, wound complexity and surveillance definitions [13,14]. The observed incidence in our cohort falls within this reported range. As a two-site regional Australian cohort, these findings may provide a contemporary benchmark for comparison with other regional and metropolitan healthcare settings. However, differences in patient populations, surgical case mix, microbiological sampling practices and infection surveillance definitions should be considered when making such comparisons.

In our cohort, infected cases had a markedly prolonged LOS compared with non-infected cases. However, because infection may occur during admission, this association likely reflects both prolonged hospitalisation resulting from infection and the greater opportunity for infection to occur during longer admissions.

Our microbiology profile was dominated by *S. aureus* and *P. aeruginosa*. This finding is biologically plausible given the established role of *S. aureus* in surgical site infection and skin and soft tissue disease [9], while *P. aeruginosa* is a recognised pathogen in chronic wounds and healthcare-associated infection [10]. Prior graft-focused studies have demonstrated variability in pathogen distribution depending on wound type and surgical setting. Unal et al. reported that infection-related graft loss occurred more frequently in vascular ulcers and burn wounds, with *P. aeruginosa* being the most commonly implicated organism [13]. Abdulmughni et al. also described high postoperative infection rates following split-thickness skin grafting with a predominance of Gram-negative organisms including *P. aeruginosa* and Klebsiella species [14]. In contrast, our cohort demonstrated a lower overall microbiologically confirmed infection incidence and was dominated by *S. aureus*, with *P. aeruginosa* representing a substantial minority of infections. Differences in case mix, wound complexity and surveillance definitions may account for this variation.

The microbiological findings provide contemporary data regarding the pathogens associated with postoperative skin graft infections within our health service and may assist clinicians when considering likely causative organisms in patients with suspected graft infection. However, empiric antimicrobial selection should continue to be guided by local antimicrobial susceptibility patterns, individual patient risk factors, infection severity and established antimicrobial stewardship principles. Our findings should therefore be viewed as complementary to, rather than a replacement for, existing therapeutic guidelines. The nested antimicrobial audit demonstrated high rates of peri-operative prophylaxis and frequent postoperative antibiotic prescribing, most commonly short courses of cephalexin. Postoperative antibiotics were commonly prescribed, while microbiological sampling was undertaken in 36.0% of encounters in the nested audit cohort. Directed therapy following culture results and infectious diseases consultation was infrequently documented. These findings suggest that culture-directed treatment represented a relatively small proportion of antimicrobial prescribing within the nested audit cohort.

Routine wound cultures are not recommended for all postoperative skin graft infections and are generally reserved for clinically significant infections, treatment failure, systemic illness, atypical presentations or situations in which microbiological results are expected to alter clinical management. Consequently, microbiological specimens were obtained selectively rather than routinely, introducing the potential for selection bias. The organism distribution reported in this study therefore reflects microbiologically confirmed infections and may over-represent pathogens associated with more severe or clinically complex presentations. These findings should not be interpreted as representing the complete microbiological spectrum of all postoperative skin graft infections. 

Postoperative antibiotic prescribing was not associated with reduced infection in this cohort; however, the analysis was limited by small sample size and low event rates. Because postoperative antibiotic prescribing was determined by clinician judgement rather than random allocation, the comparison is also susceptible to confounding by indication. Patients prescribed antibiotics may have had greater wound complexity, contamination or other clinical characteristics associated with an inherently higher risk of infection, limiting causal interpretation of the observed association.

Microbiological sampling and infectious disease involvement were more common in infected cases and are likely to reflect escalation of care rather than factors contributing to infection. These findings identify potential areas for further evaluation from an antimicrobial stewardship perspective, including microbiological sampling where clinically indicated and the use of culture results to support targeted antimicrobial therapy. Although reducing unnecessary routine postoperative antibiotic prescribing may represent an important stewardship goal, our study was underpowered to detect clinically meaningful differences in infection risk and should not be interpreted as evidence for or against the effectiveness of postoperative antibiotic therapy.

Polymicrobial infection was relatively uncommon, occurring in 7 of 66 (10.6%) microbiologically confirmed infections—five involved two organisms and two involved three organisms. This finding demonstrates that although most postoperative graft infections in this cohort were monomicrobial, a small proportion involved multiple pathogens. The presence of polymicrobial infections should also be considered when interpreting organism-specific frequencies, as each organism isolated from a polymicrobial episode was counted separately.

The role of peri-operative antibiotic prophylaxis in skin grafting remains uncertain. A systematic review and meta-analysis of elective skin graft procedures reported no statistically significant reduction in SSI with prophylactic antibiotics, suggesting limited utility for routine prophylaxis in low-risk elective cases [11]. Australian Therapeutic Guidelines: Antibiotic recommendations state that routine prophylaxis is not required for clean skin surgery, including skin grafting, and should be reserved for selected high-risk situations such as contaminated wounds or patients with specific risk factors for infection or adverse outcomes [12]. However, international guideline recommendations are not uniform and may differ according to wound classification, patient risk factors and local epidemiology. These differences reflect variation in the available evidence, healthcare settings and antimicrobial resistance patterns.

In the patient-clustered analysis, full-thickness grafts to the nose demonstrated a lower observed incidence of infection whereas the previously observed association for full-thickness facial grafts was no longer statistically significant. Higher observed infection incidence was identified among several split-thickness graft categories involving the foot, toe and extensive granulating areas. These findings should be interpreted cautiously because subgroup event counts were small, confidence intervals were wide and important patient- and procedure-level confounders were unavailable for adjustment. Potential differences in vascular supply, wound characteristics, graft size, operative technique and postoperative management may contribute to these observations. However, these factors were not measured and therefore cannot be established as explanations for the observed associations. The findings should consequently be considered hypothesis-generating and require confirmation in larger prospective studies.

Reported infection rates after skin surgery vary by setting, case mix and surveillance definitions. In dermatologic surgery systematic review data suggest SSI rates are generally low but higher among selected populations, particularly immunosuppressed patients and those with diabetes [5]. Although our cohort included a broader reconstructive case mix and used a culture-confirmed case definition, the overall rate observed is consistent with a clinically meaningful infection burden in inpatient grafting populations.

This study has several limitations. Firstly, its retrospective observational design relied on existing clinical documentation and a case definition requiring both clinician diagnosis and microbiological confirmation. No predefined postoperative surveillance interval was used and infections were identified during the available postoperative follow-up when clinically documented as being related to the graft procedure. This approach prioritised diagnostic specificity but may have resulted in inconsistent follow-up and likely underestimated the true incidence of postoperative skin graft infection compared with studies using standardised Centers for Disease Control and Prevention (CDC) or European Centre for Disease Prevention and Control (ECDC) surgical site infection definitions and surveillance periods. In addition, because data were extracted retrospectively from routinely collected clinical records, the study is susceptible to information bias arising from incomplete, inaccurate or inconsistently documented clinical information, which may have resulted in misclassification of exposures or outcomes. Furthermore, because microbiological sampling was performed selectively rather than routinely, the reported organism distribution reflects microbiologically confirmed infections and may over-represent pathogens associated with more severe or clinically complex presentations.

Secondly, although the graft type and anatomical site analyses accounted for repeated procedures using robust standard errors clustered at the patient level, the number of infection events was limited and several graft subgroups contained sparse data. Consequently, estimates for some associations were imprecise with wide confidence intervals and should be interpreted as exploratory. The reference category for graft type–site analyses comprised a heterogeneous group of small split-thickness grafts to other anatomical sites. As such, relative risks should be interpreted as comparisons with this residual category rather than with a single anatomically uniform comparator.

Thirdly, demographic variables including age/date of birth and sex were not included in the predefined dataset, preventing description of the age, sex and paediatric/adult distribution of the cohort. In addition, several important patient- and procedure-level variables recognised to influence surgical site infection risk (including diabetes mellitus, smoking status, immunosuppression, nutritional status, vascular disease, wound classification, graft size, wound contamination, indication for grafting and peri-operative management) were not collected as part of the predefined dataset and were therefore not available for analysis as covariates. Consequently, multivariable adjustment for these potential confounders was not possible and the observed associations between graft type/anatomical site and infection may partly reflect differences in underlying patient, wound or procedural characteristics. These findings should therefore be interpreted as exploratory associations rather than independent causal risk factors.

Finally, sparse event numbers across several subgroup analyses resulted in unstable estimates, wide confidence intervals and perfect prediction in some regression models. The hospital encounter-level antimicrobial prescribing audit was descriptive in nature and was restricted to a nested subset of the overall cohort. The nested antimicrobial audit comprised 119 episodes involving 116 individual patients. For analyses requiring peri-operative antimicrobial data, five patients were excluded because the relevant handwritten anaesthetic medication charts could not be reliably interpreted, leaving 111 patients with evaluable documentation. The exclusion of these five patients highlights the limitations of retrospective studies that rely on handwritten clinical records and underscores the value of complete, legible electronic medication documentation for clinical audit, antimicrobial stewardship evaluation and retrospective research. Although selection was not contingent on infection status, antimicrobial exposure, microbiological sampling or graft failure, the non-random selection of the nested audit cohort may have introduced selection bias and the nested cohort may not fully represent antimicrobial prescribing across the overall cohort. These findings should therefore be interpreted as a hospital encounter-level description of antimicrobial prescribing practices within the nested audit cohort rather than as representative of all skin graft procedures or evidence of optimal antimicrobial management.

## 5. Conclusions

Microbiologically confirmed postoperative skin graft infection occurred in 6.8% of procedures and was associated with substantially longer hospitalisation. *Staphylococcus aureus* and *Pseudomonas aeruginosa* were the most frequently identified pathogens among microbiologically confirmed infections. This study provides contemporary data on the incidence and microbiology of postoperative skin graft infection in a regional Australian cohort, alongside a descriptive assessment of antimicrobial prescribing practices in a nested audit cohort. The higher observed infection incidence in some graft type/anatomical-site categories, particularly split-thickness grafts involving the foot and toe, may help identify procedural groups warranting closer postoperative surveillance and further investigation of targeted preventive strategies. However, these findings should be interpreted as exploratory given the study’s retrospective design, lack of multivariable adjustment and other methodological limitations. In particular, the present study does not establish that targeted antimicrobial prophylaxis would reduce infection in these groups. Prospective studies incorporating comprehensive adjustment for patient- and procedure-level confounders are required before graft site-specific prophylaxis or other preventive interventions can be recommended.

## Figures and Tables

**Table 1:** Study population and infection incidence.

Parameter	Value
Individual patients	770
Total procedures	977
Microbiologically confirmed infections	66
Microbiologically confirmed infection incidence	6.8% (95% CI: 5.3–8.5%)
Study period	1st July 2021–13th August 2024

**Table 2:** Infection incidence and length of stay.

Measure	Numerator/Value	Denominator/Comparator	Result
Postoperative skin graft infection	66	977 procedures	6.8% (95% CI 5.3–8.5%)
LOS, infected cases	34 days (13–52)	Median	*p* < 0.001 vs. non-infected
LOS, non-infected cases	3 days (0–13)	Median	*p* < 0.001 vs. infected

**Table 3:** Graft type–site associations with microbiologically confirmed infection.

Graft Category	Relative Risk	95% CI	*p* Value	Interpretation
Small SSG Other Site	Reference	—	—	Reference category
Full-thickness graft to nose	0.093	0.013–0.688	0.020	Lower observed incidence
Full-thickness graft to face	0.205	0.028–1.502	0.119	No statistically significant association
Small split skin graft of foot	3.107	1.430–6.749	0.004	Higher observed incidence
SSG of extensive granulating area	4.439	1.421–13.862	0.010	Higher observed incidence
Small split skin graft of toe	5.548	1.315–23.407	0.020	Higher observed incidence

**Table 4:** Microbiological isolates.

Organism	*n*	% of All Procedures (*n* = 977)	95% CI	% of Infected Procedures (*n* = 66) *
*Staphylococcus aureus*	31	3.2%	2.2–4.5%	47.0%
*Pseudomonas aeruginosa*	15	1.5%	0.9–2.5%	22.7%
*Escherichia coli*	7	0.7%	0.3–1.5%	10.6%
Group B Streptococci	5	0.5%	0.2–1.2%	7.6%
*Acinetobacter baumannii*	1	0.1%	0.0–0.6%	1.5%
*Enterobacter cloacae*	2	0.2%	0.0–0.7%	3.0%
*Enterococcus faecium*	2	0.2%	0.0–0.7%	3.0%
*Granulicatella adiacens*	1	0.1%	0.0–0.6%	1.5%
*Haemophilus influenzae*	1	0.1%	0.0–0.6%	1.5%
*Morganella morganii*	2	0.2%	0.0–0.7%	3.0%
*Proteus mirabilis*	2	0.2%	0.0–0.7%	3.0%
Group A Streptococcus	4	0.4%	0.1–1.0%	6.1%
Group D Streptococcus	1	0.1%	0.0–0.6%	1.5%
*Yokenella regensburgei*	1	0.1%	0.0–0.6%	1.5%

* Percentages of infected procedures are calculated using the total number of microbiologically confirmed infected procedures (*n* = 66) as the denominator. Procedures with polymicrobial infection contributed to the count for each organism isolated and thus are not mutually exclusive and do not sum to 100%.

**Table 5:** Antimicrobial prescribing practices and infection outcomes in the nested audit cohort.

Variable	Value	Denominator	Comparative Statistic	Interpretation
Peri-operative prophylaxis	99	111	89.2%	Commonly used
Postoperative antibiotics	76	111	68.5%	Frequently prescribed
Microbiological sampling	40	111	36.0%	Performed in minority
Directed therapy	12	111	10.8%	Uncommon
ID consultation	8	109 *	7.3%	Uncommon
Median length of therapy	5 days	IQR 0–5	Not applicable	Short-course therapy common
Infections in follow-up cohort	7	106 *	6.6%	Low event count
Post-op antibiotics vs. no post-op antibiotics	4/73 vs. 3/33	106 *	RR 0.60 (95% CI 0.14–2.54); *p* = 0.675	No observed association; underpowered
Infection in sampled vs. non-sampled patients	6/39 vs. 1/67	106 *	RR 10.31 (95% CI 1.29–82.50); *p* = 0.0096	Escalation of care
Infection in patients with vs. without ID consultation	3/8 vs. 4/97	105 *	RR 9.09 (95% CI 2.45–33.77); *p* = 0.0090	Escalation of care

* The nested audit comprised 119 episodes involving 116 individual patients. Peri-operative antimicrobial data were evaluable for 111 patients; five patients were excluded from analyses requiring anaesthetic medication chart data because the relevant handwritten charts could not be reliably interpreted. Denominators vary further for individual variables because of missing data.

## Data Availability

The data supporting the findings of this study contain confidential patient information and are available from the corresponding author upon reasonable request, subject to institutional governance approval.

## Abbreviations

The following abbreviations are used in this manuscript:

FTSG	Full-thickness skin graft
ID	Infectious diseases
LOS	Length of stay
MRSA	Methicillin-resistant *Staphylococcus aureus*
RR	Relative risk
SG	Skin graft
SSI	Surgical site infection
SSG	Split-thickness skin graft
VRE	Vancomycin-resistant *Enterococcus*
